# The structure of a major surface antigen SAG19 from *Eimeria tenella* unifies the *Eimeria* SAG family

**DOI:** 10.1038/s42003-021-01904-w

**Published:** 2021-03-19

**Authors:** Nur Zazarina Ramly, Samuel R. Dix, Sergey N. Ruzheinikov, Svetlana E. Sedelnikova, Patrick J. Baker, Yock-Ping Chow, Fiona M. Tomley, Damer P. Blake, Kiew-Lian Wan, Sheila Nathan, David W. Rice

**Affiliations:** 1 Department of Molecular Biology and Biotechnology, Krebs Institute for Biomolecular Research, The University of Sheffield, Sheffield, UK; 2 Faculty of Science and Technology, Universiti Sains Islam Malaysia, Nilai, Negeri Sembilan Malaysia; 3 Faculty of Science and Technology, Universiti Kebangsaan Malaysia, Bangi, Selangor Malaysia; 4 Royal Veterinary College, Hertfordshire, UK

**Keywords:** Parasite immune evasion, X-ray crystallography

## Abstract

In infections by apicomplexan parasites including *Plasmodium*, *Toxoplasma gondii*, and *Eimeria*, host interactions are mediated by proteins including families of membrane-anchored cysteine-rich surface antigens (SAGs) and SAG-related sequences (SRS). *Eimeria tenella* causes caecal coccidiosis in chickens and has a SAG family with over 80 members making up 1% of the proteome. We have solved the structure of a representative *E. tenella* SAG, EtSAG19, revealing that, despite a low level of sequence similarity, the entire *Eimeria* SAG family is unified by its three-layer αβα fold which is related to that of the CAP superfamily. Furthermore, sequence comparisons show that the *Eimeria* SAG fold is conserved in surface antigens of the human coccidial parasite *Cyclospora cayetanensis* but this fold is unrelated to that of the SAGs/SRS proteins expressed in other apicomplexans including *Plasmodium* species and the cyst-forming coccidia *Toxoplasma gondii*, *Neospora caninum* and *Besnoitia besnoiti*. However, despite having very different structures, Consurf analysis showed that *Eimeria* SAG and *Toxoplasma* SRS families each exhibit marked hotspots of sequence hypervariability that map to their surfaces distal to the membrane anchor. This suggests that the primary and convergent purpose of the different structures is to provide a platform onto which sequence variability can be imposed.

## Abstract (web summary)

Ramly, Dix et al. report the first high-resolution crystal structure of a major surface antigen (SAG) from *Eimeria tenella*, a parasite that infects poultry. This structure represents the prototype for many *Eimeria* SAGs and provides insight into the function of these surface proteins.

## Introduction

*Eimeria* are economically important apicomplexan parasites that cause coccidiosis in chickens; infection damages the gastrointestinal tract leading to nutrient malabsorption, weight loss and, in severe cases, haemorrhage and death^1–3^. A key feature that enables *Eimeria* to infect and multiply in chickens is its complex life cycle that encompasses both sexual and asexual stages of development^4^. Although the duration of an individual *Eimeria* infection is relatively short (~10–16 days duration in total, depending on the infecting *Eimeria* species), coccidiosis can be serious if chickens ingest large numbers of parasites, and pathology is associated with the rapid intracellular replication of parasites that severely damages the gut epithelium^5^. Seven species of *Eimeria* are recognised to infect chickens with three species (*E. tenella*, *E. maxima* and *E. acervulina*) considered to be most important due to their high prevalence and disease potential^6^. The global economic burden of coccidiosis is estimated to exceed £2 billion per annum through a combination of losses associated with infection and the costs of control^7^.

Analysis of apicomplexan genomes, including those of several *Plasmodium* species and *Toxoplasma gondii*, has identified the presence of gene families arranged in tandem arrays that encode cysteine-rich antigenic proteins that are expressed on the surface of invasive parasite stages (variously termed surface antigens, SAGs and SAG-related sequences, SRS). It has been suggested that these are important in mediating parasite interactions with the host immune system and in initial attachment to the host cells^8–11^. Genome and mRNA sequencing studies in *E. tenella* identified over 80 different *sag* genes^12^. Based on predicted protein sequence similarity the *E. tenella* members of the SAG family (EtSAGs) were divided into three sub-families (SAG^A^, SAG^B^ and SAG^C^) that share less than 5% overall sequence identity^12^. In addition, a further 20 regions of the *E. tenella* genome were identified as encoding *sag* pseudogenes^12^. These EtSAGs comprise a cysteine-rich ectodomain of ~300 residues, including an N-terminal signal sequence and a site at the C-terminus for a glycosylphosphatidylinositol (GPI) anchor for membrane attachment^11^. In *E. tenella*, these different proteins, which comprise some 1% of the proteome^12^, are differentially expressed during the invasive sporozoite and multiple merozoite stages of the parasite’s life cycle and a subset were shown to cause a pro-inflammatory response in cultured avian macrophages^11–13^.

Comparison of sequences of *Eimeria* SAGs with SAGs/SRS from *Toxoplasma* and *Plasmodium* species failed to produce a convincing alignment, suggesting that they have a different molecular structure^12^ consistent with a predicted model for the structure of *E. tenella* SAG1 suggesting a fold related to cysteine-rich secretory proteins (CRISPs), antigen 5 and pathogenesis-related 1 proteins (CAP) with low-level sequence similarities^14^. In this paper, we report an experimental three-dimensional structure for a representative of the SAG^B^ sub-family, *E. tenella* SAG19 (EtSAG19) (Uniprot: Q70CD0) at 1.32 Å resolution. The structure reveals that the fold of EtSAG19 comprises a three-layer αβα sandwich that is distinct from that of the SAGs/SRS of *Toxoplasma* and *Plasmodium*^15,16^. Moreover, analysis of the EtSAG19 structure and patterns of sequence similarity shows that this fold is also adopted by members of the *Eimeria* SAG^A^ and SAG^C^ sub-families, thereby unifying the entire *Eimeria* SAG family despite their extensive sequence differences. Further structure comparisons confirmed that the fold of the *Eimeria* SAGs is related to that of the CAP superfamily that include a range of toxins from insects, reptiles and plants suggesting some functional conservation of this domain across these disparate groups of proteins.

## Results

### The overall structure of EtSAG19

**Fig. 1:**
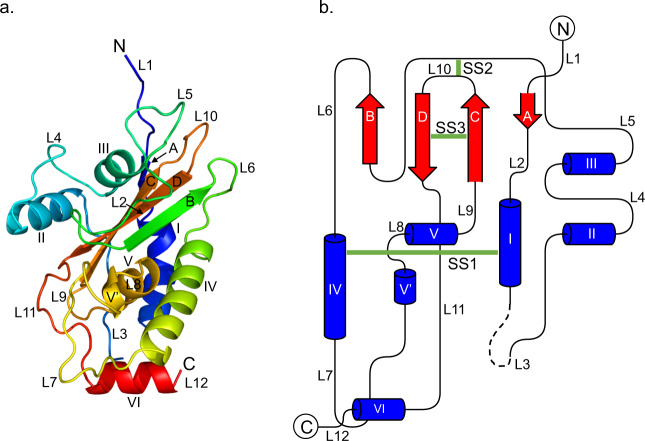
3D structure of *E. tenella* SAG19. **a** Chainbow representation of the 3D structure of EtSAG19 indicating the structure consists of four β-strands (A–D), six α-helices (I–VI) and a single 3^10^ helix (V’). **b** 2D topology map of EtSAG19 with the N- and C-termini labelled. The locations of the three disulphide bonds are shown by green bars.

The structure of *E. tenella* SAG19 was solved by sulphur-SAD to 1.32 Å to reveal that the protein adopts an αβα sandwich fold, and providing an experimental three-dimensional model for the protein (Fig. 1). This fold is completely unrelated to that of the dimeric *T. gondii* SAG1 (1KZQ and 2JKS)^15,17^ and SAG2 (2WNK)^18^ families and related SRS domains (6E62) found in *P. falciparum*^16^, all of which fold into two β-sheet sandwich domains, explaining the uncertainty surrounding initial attempts to align the sequences of EtSAG19 to these proteins. The structure of EtSAG19 is thus currently unique among SAGs from apicomplexan parasites. EtSAG19 comprises a four-stranded, anti-parallel, β-sheet surrounded by six α-helices with an additional 3^10^ helix and 12 loops, 10 of which connect regions of regular secondary structure together with two additional loops to the N- and C-terminus. The loop regions in EtSAG19 are extensive and account for ~50% of the entire structure with some of the loops being large with, for example, 20 residues in the loop between helix III and strand B (L5) (Fig. 1a, b and Supplementary Fig. 1). As predicted, given its cysteine content, EtSAG19 contains three disulphide bonds, SS1 (connecting C67 and C152), SS2 (connecting C126 and C213) and SS3 (connecting C207 and C222). The structure also contains three cis peptides, two of which involve proline (E105-P106 in loop L4 that links helix II to helix III and, A184-P185 in loop L7 leading to the 3^10^ helix, helix V’). The third cis peptide is more unusual in that it is a rare non-proline cis peptide between I225 and D226 in loop L11 linking strand D to helix VI, forming a tight turn in the structure.

### Conservation of sequence and structure between the EtSAGs provides a unified model for the entire family

Following assembly of the *E. tenella* genome sequence, all of the predicted EtSAG coding sequences were grouped into three sub-families for which 60 (SAG^A^), 28 (SAG^B^) and 1 (SAG^C^) members could be identified (Supplementary Data 1)^12^. The determination of the EtSAG19 structure, a representative of the SAG^B^ sub-family, facilitated a more detailed examination of all the predicted EtSAG sequences. Of the 60 and 28 *E. tenella* sequences in the SAG^A^ and SAG^B^ sub-families, seven and two members, respectively, were excluded from a structure-based sequence alignment on the basis that the resulting patterns of insertions and deletions suggested they were outliers. These exclusions served to simplify the subsequent analysis and, given the incomplete status of the genome assembly, avoided potential uncertainty associated with the possibility that these sequences might be those of pseudogenes.

**Fig. 2:**
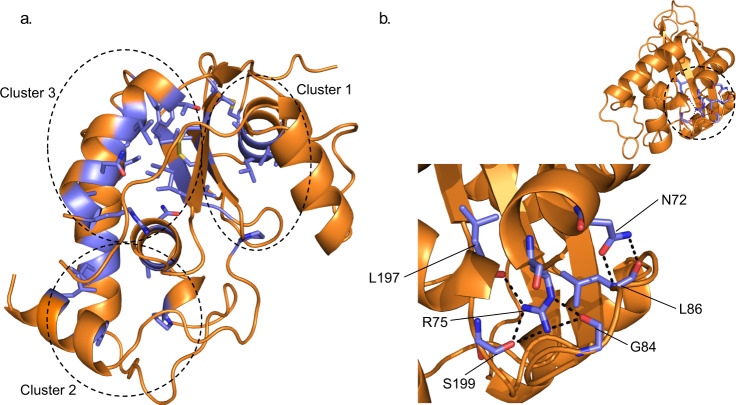
Structural organisation of the conserved residues found within the SAG^B^ sub-family members. **a** 3D location of the 37 conserved residues (highlighted in blue) identified within the SAG^B^ sub-family mapped onto EtSAG19. The conserved residues can be grouped into three clusters (clusters 1, 2 and 3) as indicated by the dotted circles. **b** Within cluster 3 two residues, N72 and R75, are of particular interest due to their polar and buried nature. N72 and R75 are involved in an intricate hydrogen bonding network with four surrounding residues (Supplementary Table 1).

The alignment of the remaining 26 SAG^B^ members against the EtSAG19 structure identified that, excluding the regions covered by the signal sequence and GPI anchor, 37 (~20%) of the residues were strongly conserved (defined as being identical in at least 95% of the sequences) (Supplementary Fig. 1). Analysis of the structure showed that these conserved residues are located within the buried core of the protein, and broadly lie in three clusters, one (cluster 1) on one face of the central β-sheet with the remaining two clusters (clusters 2 and 3) found on the opposite face (Fig. 2a). Cluster 1 consists of 12 largely hydrophobic residues that serve to pack αII and αIII against the β-sheet and comprise the disulphide SS2 (joining L5 the loop between αIII and βB to L10 the loop between βC and βD), G120 and G139 on L5 the loop between αIII and βB, G216 on the loop L10 between βC and βD, I114, A115, L118 and A119 from αIII, V141 from βB, T210 from βC and P227 that is located on the loop L11 between βD and αVI. A notable feature of cluster 1 is the close approach (3.0 Å) of one of the carboxyl oxygens, OD1 of D218 to SG of C126 from SS2 implying that these atoms may be involved in a hydrogen bond. This suggests that the carboxyl is protonated, an interpretation that is supported by the common substitution of this residue to asparagine in some SAG^B^ sub-family members.

Cluster 2 is responsible for maintaining the interaction between helices αIV, αV, V’ and αVI. The eight conserved residues involved in this cluster include A162, G163 and L164 of αIV, P185 of V’, A191 and A195 of αV and Y240 and L243 of αVI. Cluster 3 is the most extensive involving a network of 17 residues responsible for maintaining the packing of αI, the loop L3 between αI and αII and αIV to the central β-sheet. This network includes the remaining two disulphides SS1 (joining αI and αIV) and SS3 (connecting βC and βD), T64, L68, N72, L74 and R75 of αI, F150 located in loop L6 between βB and αIV, L155 and I156 of αIV, N193 of αV, L140, I142 and F144 of βB and L220 of βD.

In contrast to the conservation of buried residues, there are no conserved residues on the surface of the SAG^B^ sub-family. Hence, this pattern of sequence conservation implies that the SAG^B^ members do not have an enzymatic role in *Eimeria* and that the sequence conservation presents a constraint necessary for maintaining the overall 3D fold of the sub-family. Of the residues that are buried in the structure of EtSAG19, the conservation of N72 and R75 at one end of cluster 3 as part of an NxxR motif in members of the SAG^B^ sub-family is particularly noteworthy given the polar nature of their sidechains. Analysis of the structure shows that these sidechains participate in an intricate network of hydrogen bonds (Supplementary Table 1 and Fig. 2b) that help to pin the extended loop L3 found between αI and αII to αI and the C-terminus of αV.

While the sequence similarity between the SAG^B^ and SAG^A^ sub-families is low (~5%), inspection of the 53 aligned sequences of the SAG^A^ sub-family members against each other and against the SAG^B^ sub-family (Supplementary Data 2) revealed that ten residues are strongly conserved including the N72xxR75 motif, the six cysteines in the three disulphides, G139 and A195. The location of these residues in one or other of the three clusters implies that both SAG^B^ and SAG^A^ members share the same 3D structure. As expected, and consistent with the structure of EtSAG19, the positions of the insertions and deletions between members of the SAG^B^ and SAG^A^ families fall in the loops between elements of secondary structure (Supplementary Fig. 2).

In previous work on *E. tenella*, it has been established that proteins, now assigned to the SAG^A^ sub-family, undergo cleavage at a conserved RRL motif^14^ not found in SAG^B^ sub-family members. Comparison of the sequences shows that in SAG^A^ this motif is part of the largest insertion between SAG^A^ and SAG^B^ members consisting of approximately 30 residues. Analysis of the EtSAG19 structure reveals that this insertion is found within L10 and must form part of a surface-exposed extension in the structure between βC and βD, which, in EtSAG19, are directly linked by a short connection of only six residues (Supplementary Fig. 2). Since, following cleavage, cysteines on either side of the cleavage point remain connected by a disulphide bond (equivalent to C207 and C222 of SS3 in EtSAG19) and further, since βD lies in the centre of the beta sheet, we suggest that the fold of the protein will likely be maintained in SAG^A^ members that are susceptible to cleavage.

Extension of the sequence analysis to include the third SAG sub-family, SAG^C^, necessitated the inclusion of the more extensive repertoire of SAG^C^ members as only a single SAG^C^ sequence is found in *E. tenella* (ETH_00001975). The two *Eimeria* species with the largest number of SAG^C^ sub-family members are *E. brunetti* (38 members) and *E*. *mitis* (27 members). We elected to carry out an initial alignment and analysis including only *E. brunetti* SAG^C^ members (Supplementary Data 3), as *E. mitis* contains more pseudogenes. Across all three sub-families of *Eimeria* SAGs, seven residues (~2%) are strongly conserved including, R75 of the N72xxR75 motif, the four cysteines belonging to SS2 and SS3 together with P234 and F235. This conservation confirms that the fold of SAG^C^ is essentially the same as that of SAG^A^ and SAG^B^, but with major insertions and deletions particularly in loops L3, L4, L5 and L10 that represent hotspots for sequence change. The conservation of this underlying similarity in the fold unifies all the SAGs within one family and therefore, we propose that, rather than sub-dividing these proteins into three different sub-families, it is more appropriate to consider them as being members of a single family with widely divergent sequences. Comparing the findings from this analysis with the aligned sequences of SAG^C^ members from *E. mitis* (Supplementary Data 4), the pattern of sequence conservation seen in *E. brunetti* SAG^C^s, including the strong conservation of the NxxR motif, is the same. The only exception to this is that in 8 of the 27 *E. mitis* SAG^C^ the C-terminus is truncated, such that the GPI anchor would be lost, indicating these might be pseudogenes or as yet incomplete sequences.

### EtSAG19 belongs to the CAP superfamily

**Fig. 3:**
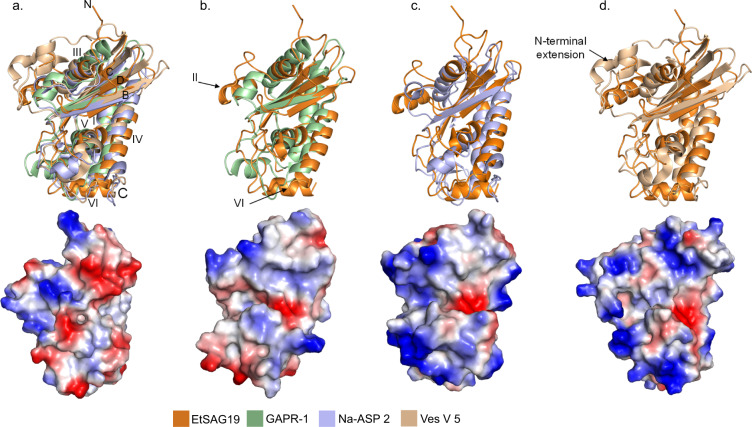
Structural alignments of EtSAG19 with other CAP superfamily representatives and their respective electrostatic surfaces. **a** (Top) Alignment of GAPR-1 (green), Na-ASP 2 (blue) and Ves V 5 (brown) with EtSAG19 (orange) shows the structural conservation of three β-strands (B–D) and four α-helices (I, III, IV and V). (Bottom) Electrostatic surface of EtSAG19. **b** (Top) Alignment of GAPR-1 and EtSAG19 highlighting the extra helices (II and VI) identified in EtSAG19 that are not found in the other CAP protein. (Bottom) Electrostatic surface of GAPR-1. **c** (Top) Alignment of Na-ASP 2 and EtSAG19 indicating the C-terminal extension seen in Na-ASP 2. (Bottom) Electrostatic surface of Na-ASP 2. **d** (Top) Alignment of Ves V 5 and EtSAG19 indicating the N-terminal extension of Na-ASP 2 and its longer β-strands. (Bottom) Electrostatic surface of Ves V 5. The colours of the different proteins are shown in the included key and all views are from the same orientation. For the electrostatic surfaces, the ranges of kbT/e– represented by dark red to dark blue that are set by the programme defaults are as follows: EtSAG19 (–67.9 to 67.9), GAPR-1 (–71.4 to 71.4), Na-ASP 2 (–56.7 to 56.7) and Ves V 5 (–72.2 to 72.2).

To identify other proteins with a similar fold to EtSAG19, the structure was submitted to the Dali server^19^. This revealed that, despite almost no sequence similarity, the fold of EtSAG19 showed a high degree of structural similarity to representatives of proteins belonging to the CAP superfamily, including, GAPR-1^20^ (Golgi-associated pathogenesis-related protein) (PDB = 1SMB, Z score = 12.0), Ves V 5^21^ (PDB = 1QNX, Z score = 10.8) and Na-ASP 2^22^ (PDB = 1U53, Z score = 10.3) as the top hits. Superposition of the structures of GAPR-1, Ves V 5 and Na-ASP 2 with EtSAG19 using LSQKAB^23^ identified 63 equivalent Cαs with RMS deviations of 1.07, 1.09 and 1.17 Å (Supplementary Table 2), respectively, despite only 8, 13, and 12% sequence identity. This confirmed the previous suggestion from earlier modelling studies that these proteins might share a related fold^14^. The main conserved elements of the structures include β-strands B, C and D of the central beta sheet, in addition, α-helices I, III, IV and V that pack against it (Fig. 3a). Structural differences between these proteins are mainly confined to the loop regions, but also include additional helices in EtSAG19 (helices II and VI) (Fig. 3b), a unique C-terminal extension in Na-ASP 2 (Fig. 3c) and the N-terminal extension and generally longer strands in Ves V 5 (Fig. 3d).

A more remote similarity, identified by Dali, can be seen between EtSAG19 and another member of the CAP superfamily, Tablysin-15 (3U3U) from *Tabanus yao* (Z score = 9.3, RMSD = 2.8 Å, 14% sequence identity). Tablysin-15 is found in the saliva of the horse fly *Tabanus yao* and is a potent inhibitor of platelet and endothelial cell function^24^. Structural analysis of tablysin-15 showed that it contains an RGD (Arg-Gly-Asp) motif that mediates binding of tablysin-15 to integrins that inhibits the adhesion of platelets to fibrinogen and endothelial cells to the matrix protein vitronectin^24^. In addition, tablysin-15 has been shown to contain a hydrophobic cleft that allows for the binding of cysteinyl leukotrienes, thought to act as an anti-inflammatory agent^24^. In tablysin-15, the leukotriene-binding pocket lies between helices H1, H3 and H4. However, analysis of the EtSAG19 structure revealed that the arrangement of the equivalent helices (HI, HIV and HV) is subtly different such that the sidechains of α-helices (H1 and HIV) are in close contact to each other resulting in the abolition of the pocket. The absence of the RGD motif and the hydrophobic lipid-binding pocket in EtSAG19 and other members of the CAP superfamily suggests that the action of EtSAG19 is not linked to the binding of a small molecule effector.

Interestingly, a structure-based sequence alignment between EtSAG19 and the CAP superfamily showed that the two residues, N72 and R75, of the NxxR motif are strongly conserved. The unusual nature of the conserved buried Arg75, including the four hydrogen bonds to main-chain carbonyl oxygen atoms, was explored more widely using the ASSAM server^25^. This comparison showed that interactions equivalent to those seen for Arg75 in EtSAG19 are present in other members of the CAP superfamily. Therefore, the conservation of N72 and the buried arginine, R75, are particularly important in preserving the overall 3D structure of the protein, and, in our view, act as a fingerprint for the fold. In addition, the two cysteines (C207 and C222) belonging to SS3 in EtSAG19 are strongly conserved across the superfamily and only absent in the human GAPR-1 protein. It is also interesting to note that despite their sequence differences, the non-proline cis peptide (I225-D226) found in EtSAG19 aligns with a cis-proline found in Ves V 5 (PDB ID: 1QNX) and Na-ASP 2 (PDB ID: 1U53). This indicates that a cis peptide in this region is an important feature critical for the maintenance of the structure within the majority of the proteins with this fold.

### The surface of the SAGs is a hotspot for sequence variation that may be functionally important

**Fig. 4:**
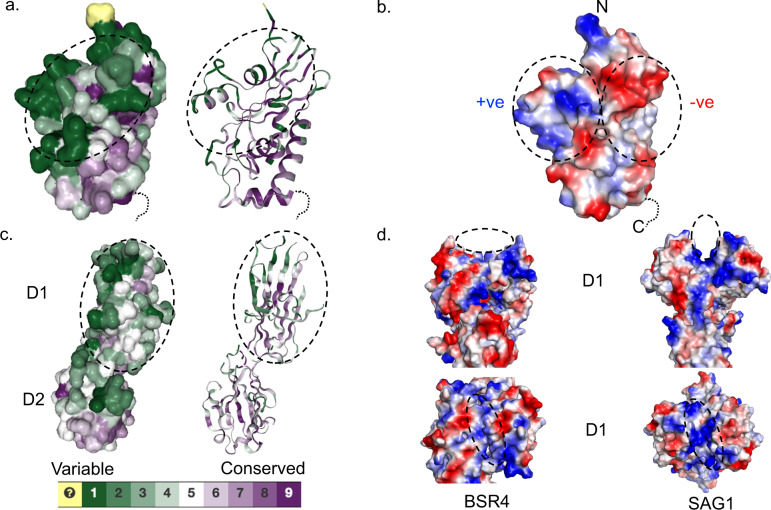
Surface conservation and electrostatic distribution found within *E. tenella* and *T. gondii* SAGs. **a** Surface (left) and cartoon (right) representation of EtSAG19 displayed using the Consurf sequence conservation colour scheme with purple indicating the most strongly conserved. This indicates that the maximal sequence variability lies on the surface of the molecule distal from the C-terminal GPI anchor. The associated cartoon diagram indicates that the conservation is located within the buried core of the protein. **b** Electrostatic representation of the surface of EtSAG19. This highlights the distinct localisation of charge to the N-terminal end of the molecule as part of which one face has a local net negative charge whilst the other is positive. **c** Surface (left) and cartoon (right) representation of *T. gondii* BSR4 monomer displayed using the Consurf sequence conservation colour scheme. This highlights the high degree of sequence variability found within the interconnecting loops of the D1 domain, distal from the C-terminal anchor, whereas the D2 domain is highly conserved. **d** Electrostatic representation of the *T. gondii* D1 domains from the BSR4 (left) and SAG1 (right) dimers. In the top view the twofold of the dimer is vertical, whereas in the lower view it is towards the reader. This shows that the maintenance of a charged cluster of residues at the dimer interface of both SAGs, which form a cleft, believed to be important for molecular interaction of their respective targets. Electrostatic ranges of kbT/e– represented by dark red to dark blue that are set by the programme defaults are as follows: EtSAG19 (–67.9 to 67.9), SAG1 (–63.6 to 63.6) and BSR4 (–61.5 to 61.5).

While it has been established that SAGs are expressed on the surface of *Eimeria* parasites^11^, it is currently unknown whether these proteins are involved in aspects of cell adhesion, invasion or in some other process^11,13^. To gain further insights into the function of the EtSAGs, the wide-ranging sequence analysis obtained by mapping the aligned SAG sequences from the *E. tenella* SAG^A^ and SAG^B^ families and the SAG^C^ sequences from *E. tenella* and *E. brunetti* onto the EtSAG19 structure was analysed using Consurf^26^. This indicated that sequence variations across the families, including the major sites of insertions and deletions, are broadly located on the surface of one face of the central β-sheet adjacent to cluster 1 in the three-dimensional structure of EtSAG19 (Fig. 4a). This patch consists of the surface-exposed residues located within the N-terminal loop, αII, the loop (L4) between αII and αIII, αIII, the loop (L5) between αIII and βB, and the loop (L10) connecting βC and βD (Fig. 4a). These residues lie remote from the C-terminal GPI anchor and therefore point outwards from the parasite plasma membrane in such a way as to be readily solvent accessible and include two regions of the EtSAG19 surface that are positively and negatively charged, respectively (Fig. 4b). However, lack of sequence conservation in the latter regions indicates that the nature of the charged surface is not conserved across all members of the EtSAG family. In addition, despite the similarity in fold between the CAP superfamily members and EtSAG19, we note that, while the surface of the former display regions that show considerable charge, the location and nature of these charged surfaces are not related to those in EtSAG19 (Fig. 3a–d).

The observation that the most major sequence changes are most likely to be distal to the membrane raised the question as to whether this hotspot for sequence variation is unique to the *Eimeria* SAGs or whether *Toxoplasma* SAGs/SRS proteins display a similar phenomenon. Analysis of the annotated *T. gondii* genome within Interpro^27^ identifies 145 proteins belonging to the *Toxoplasma* SRS family. These were used in an equivalent Consurf analysis using the structure of the *Toxoplasma* SAG, BSR4^17^. This clearly identified the interconnecting loops in the N-terminal domain (D1) of BSR4 as a hotspot for sequence variability, compared to the C-terminal domain (D2) (Fig. 4c) to which the GPI anchor is attached. Furthermore, these regions of high sequence variability within *Toxoplasma* SAG1 (25% sequence identity with BSR4) also correspond to charged surfaces within D1 (Fig. 4d) that have been implicated in the binding of sulphated proteoglycans as part of the molecular function of this family of SAGs/SRS^15,17^.

The SRS proteins are widely observed in other cyst-forming coccidial parasites including *Besnoitia besnoiti* (280 identified SRS members (Interpro family: IPR007226)) and *Neospora caninum* (233 identified SRS members (Interpro family: IPR007226)) both of which cause serious diseases of livestock^28,29^. Sequence analysis identifies that the SRS proteins of these two parasites are related to the *T. gondii*-like family, rather than the *Eimeria* SAG family. Nevertheless, a parallel Consurf analysis of SRS proteins in *B. besnoiti* and *N. caninum* to that conducted for *T. gondii* reveals that the region with maximal sequence variation again lies on the face of the D1 domain, distal from the membrane with more similarity being seen in the D2 domain, consistent with the analysis of the *T. gondii* SAGs/SRS proteins (Supplementary Fig. 3).

Conversely, analysis of the genome of the human non-cyst-forming coccidial parasite *Cyclospora cayetanensis* identified four putative protein sequences with similarities to each other and to *Eimeria* SAG19. The sequences of three of these (the fourth, OEH78676.1, consisting of 85 residues was ignored as it is essentially identical to the N-terminal signal region of gene XP_022591469.2) could be aligned with confidence to the EtSAG19 sequence and other *E. tenella* SAG representatives suggesting they share a similar fold (Supplementary Fig. 4). Further analysis of the sequence alignment of the *C. cayetanensis* and *E. tenella* SAGs identified similarities in the positions of maximal sequence variation as exemplified by the major sites of insertions and deletions, which are located predominantly on the same face of the central β-sheet and distal from the GPI anchor as seen in the *E. tenella* SAGs.

Therefore, across genera of both the cyst-forming (*Toxoplasma, Neospora, Besnoitia)* and non cyst-forming (*Eimeria* and *Cyclospora*) coccidia it is evident that hotspots of sequence variability and clusters of charged residues are located to regions of their GPI-linked surface structures that lie distal to the external surface of the plasma membrane of their respective parasites. Although the role of these patches in both *Eimeria* and *Toxoplasma* SAGs/SRS is not fully understood, their location suggests that they are likely to be engaged in direct interaction with the host cell surface.

## Discussion

The structure determination of *E. tenella* SAG19 and sequence comparisons with members of the EtSAG family have established that the fold of all these proteins is related and common to all members of the CAP superfamily. It has been proposed that members of this superfamily are involved in diverse biological activities. For example, the PR-1-related proteins from plants are thought to play a role in plant defence mechanisms through the triggering of hypersensitive reactions^30^. In addition, PR proteins are suggested to be involved in antifungal activity^31^ and systemic acquired resistance^20,32,33^. Other CAP superfamily members have been identified in a wide variety of organisms unrelated by phylogeny^22,34^ and include insect allergens of the antigen 5 family^20,35,36^, mammalian CRISPs^20,37^ including human GliPR and RTVP-1 that are expressed in human brain tumours^20,38,39^, as well as lizard or snake venoms that are reported to block ryanodine receptors or cyclic nucleotide-gated ion channels^20,40^. The intriguing finding that *Eimeria* SAGs are structurally similar to members of the CAP superfamily suggests that some aspects of their function may overlap.

In an earlier study on selected *E. tenella* SAGs, we demonstrated that these GPI-anchored proteins display variable immunogenic properties^13^. For example, *E. tenella* recombinant SAGs 2, 3, 4, 5, 12, 15, 16, 18, 19 and 23 elicited chicken humoral immune responses but SAGs 4, 5 and 12 (all SAG^A^ proteins) impaired development of host cellular-mediated immunity suggesting an involvement in the inflammatory responses observed in infected chicken caeca^13^. While the functional role of EtSAGs is not clear, a modelling study on *E. tenella* SAG1 (SAG^A^ sub-family) suggested that parasite invasion may be initiated by the attachment of sporozoites to the negatively charged proteoglycans on the surface of the host cell in a process that mirrors the putative function of *T. gondii* SAG1 and SAG3^14,15^. Furthermore, several recombinant-expressed SAG^A^ proteins were shown to bind cultured cells that suggest that attachment is a potential function of the SAG^A^ family members^12^. In contrast, SAG^B^ proteins, which are most highly expressed in second-generation merozoites, were not found to bind cultured cells, suggesting they are less likely to be implicated in attachment. Together, these data suggest that differential expression of *Eimeria* SAGs may contribute to modulating the host immune system as part of a self-defence mechanism to protect the viability of the parasite and to allow it to complete the asexual and sexual stages of its self-limiting life cycle.

Our structural analysis of *Eimeria* SAGs clearly reveals that there are at least two distinct families of apicomplexan SAGs/SRS proteins as exemplified by the *E. tenella* SAGs and *T. gondii* SRS proteins. Surface proteins in *C. cayetanensis* are structurally related to the SAGs of *Eimeria* whereas those from *B. besnoiti* and *N. caninum* are related to the SRS of *T. gondii*. Interestingly, it is the SRS fold of the cyst-forming coccidia, rather than the SAG fold, that is conserved in some SAGs of the more distantly related apicomplexan genus *Plasmodium*^16^. There are currently no examples of apicomplexan parasites containing members of both families.

Despite dramatic difference in the SAG and SRS folds, the finding that sites of sequence hypervariability within *Eimeria* SAGs lie distal to the membrane closely paralleling the situation in the *T. gondii* SAGs/SRS proteins suggests that the primary purpose of each structure is to provide a stable platform onto which sequence variability can be imposed. The fact that different genera of apicomplexan parasites have acquired divergent structures on which to display surface heterogeneity suggests that this is a feature that promotes selective advantages. For individual parasites, surface heterogeneity could potentially increase avidity of the interactions with host cell surfaces mediated by SAGs/SRS proteins in *Eimeria*^12^ and *Toxoplasma*^41^. Moreover, at a population level, expression of diverse surface structures could be a critical survival mechanism for those parasites that evade host immunity and establish chronic infections. Indeed, recent in-depth single-cell transcriptional analysis of *T. gondii* revealed previously hidden heterogeneity and sporadic/switching in expression of SAG/SRS genes, mediated by the AP21X-1 transcription factor^42^.

Although the precise roles of SAG hypervariable regions are, as yet, unknown, the structural data gleaned from the EtSAG19 structure have provided an unexpected clue as to the function of the SAGs that now needs to be explored in a series of studies focusing on their structural diversity.

### Experimental procedures

#### Cloning, over expression, crystallisation and structure determination of EtSAG19

*E. tenella* SAG19 was over-expressed in *E. coli* Rosetta gami 2 (DE3), purified and crystallised as previously described^43^. Prior to data collection, crystals were loop-mounted and placed in a cryo-solution containing 2.6 M ammonium sulphate, 0.1 M citrate buffer pH 4.5 and 20% glycerol for approximately 1 min at room temperature before they were flash cooled in liquid nitrogen. The structure was determined by exploiting the phasing power of the sulphur atoms in the three disulphides of EtSAG19 using Sulphur-SAD with data to 2.09 Å resolution collected at an X-ray wavelength of 1.7 Å at Beamline I04 at the Diamond Light Source, Oxford (Table 1). The Sulphur-SAD data were processed using the XIA2 auto processing pipeline with XDS^44,45^. A second high-resolution native dataset was collected at a wavelength of 0.9795–1.32 Å resolution on Beamline I02 (Table 1). The data were processed using XIA2 with XDS^44,45^. The crystals of EtSAG19 belong to space group I4 with a single monomer in the asymmetric unit and PHENIX Autosol^46^ was employed to determine the anomalous scattering substructure that comprised sites of sulphur atoms from the three disulphides and from a bound sulphate ion. Subsequently, a preliminary set of phases was calculated and improved by density modification^47^. The resultant electron density map was interpreted by Autobuild^46^ to give a model consisting of 185 residues of poly Ala out of the expected 211 residues in the construct (residues 46-250 of the EtSAG19 coding sequence (numbered from the full-length sequence including the signal peptide) together with a hexahistidine tag).

**Table 1:** Data collection, phasing and refinement statistics for EtSAG19.

	Native	Sulphur-SAD
*Data collection*		
Wavelength (Å)	0.9795	1.7
Space group	I4	I4
Cell dimensions		
* a*, *b*, *c* (Å)	108.1, 108.1, 37.4	108.5, 108.5, 37.6
α, β, γ (°)	90.0, 90.0, 90.0	90.0, 90.0, 90.0
Resolution (Å)	23.4–1.32 (1.34–1.32)	27.13–2.09 (2.16–2.09)
*R*_merge_	0.036 (0.579)	0.052 (0.419)
*I*/σ*I*	17.6 (1.4)	48.0 (2.8)
Completeness (%)	96.9 (75.0)	78.9 (26.1)
Redundancy	5.5 (2.6)	24.1 (3.7)
Total reflections	270,326 (7356)	247,966 (904)
Unique reflections	49,236 (2869)	10,310 (243)
CC_1/2_	0.999 (0.535)	0.999 (0.752)
Wilson B factor (Å^2^)	14.8	29.405
Anomalous completeness (%)	N/A	77.6 (13.5)
Anomalous multiplicity	N/A	12.5 (2.5)
Anomalous correlation	N/A	0.174 (0.215)
Anomalous slope	N/A	1.023
*Refinement*		
Resolution (Å)	23.41–1.32	
No. reflections	49236	
*R*_work_/*R*_free_	0.165/0.188	
No. atoms		
Protein	1448	
Ligand/ion	10	
Water	259	
*B*-factors		
Protein	18	
Ligand/ion	42	
Water	32	
R.m.s. deviations		
Bond lengths (Å)	0.0109	
Bond angles (°)	1.596	
Molprobity score	0.98 (99th percentile, 1.32 ± 0.25 Å)	
PDB ID	6ZZB	

#### Structure refinement and analysis

The initial model was refined using the 1.32 Å resolution data in *REFMAC*^48^. The structure was improved by rebuilding in COOT^49^ with the addition of water molecules by *ARP/WARP*^50^ interspersed with further cycles of refinement by REFMAC^48^. The final model comprised 185 residues belonging to EtSAG19 together with 259 water molecules, and two sulphate ions. In the corresponding final electron density map, the density for the main chain of the residues included in the model is unambiguous (Supplementary Fig. 5). Weak density can be seen for four further residues representing amino acids 77–80, but, since the interpretation of this region was ambiguous, these residues were not included in the model. The model includes multiple conformations for five residues all of which are located in loop regions (Ser76, Ser121, Ser128, Asn153 and Ser170). No density can be seen for the first eight amino acid residues at the N-terminus and the last four residues at the C-terminus together with residues from the hexahistidine tag. The final model had 0% Ramachandran outliers, with 98.9% of the residues having favoured angles. In addition, the model contains 0% poor rotamers with 96.9% of the residues displayed favoured rotamers. We note that one of the most major deviations from ideality in the final model is associated with the C-N-CA bond angle of ASP 226, the residue involved in the non-proline CIS peptide.

### Statistics and reproducibility

Model validation was carried out using Molprobity^51^ and final refinement statistics are presented in Table 1. Sequence alignments are presented using Jalview^52^. Structural similarity searches were carried out using the Dali web server^19^ leading to the finding that the SAG fold is closely related to members of the CAP superfamily with Dali Z-scores of ~10 where scores greater than 2.0 are generally regarded as being significant (further details of the Z score can be found in Holm et al.^19^).

### Reporting summary

Further information on research design is available in the Nature Research Reporting Summary linked to this article.

## Supplementary information

Supplementary Information

Description of Additional Supplementary Files

Supplementary Data 1

Supplementary Data 2

Supplementary Data 3

Supplementary Data 4

Reporting Summary

## Data Availability

Images used in data processing are available upon request. The atomic coordinates and structure factors for EtSAG19 are available at the PDB with accession code 6ZZB.
