# Supplementary material for: The structure of a major surface antigen SAG19 from Eimeria tenella unifies the Eimeria SAG family
Source: Commun Biol. 2021 Mar 19;4:376. doi: 10.1038/s42003-021-01904-w (PMC7979774; doi:10.1038/s42003-021-01904-w)
Supplement: Supplementary file 1 — Supplementary Information [file 42003_2021_1904_MOESM1_ESM.pdf]

The structure of a major surface antigen SAG19 from *Eimeria tenella* unifies the *Eimeria* SAG family

Nur Zazarina Ramly, Samuel R. Dix, Sergey N. Ruzheinikov, Svetlana E. Sedelnikova, Patrick J. Baker, Yock-Ping Chow, Fiona M. Tomley, Damer P. Blake, Kiew-Lian Wan, Sheila Nathan, and David W. Rice

Supplementary figures and tables

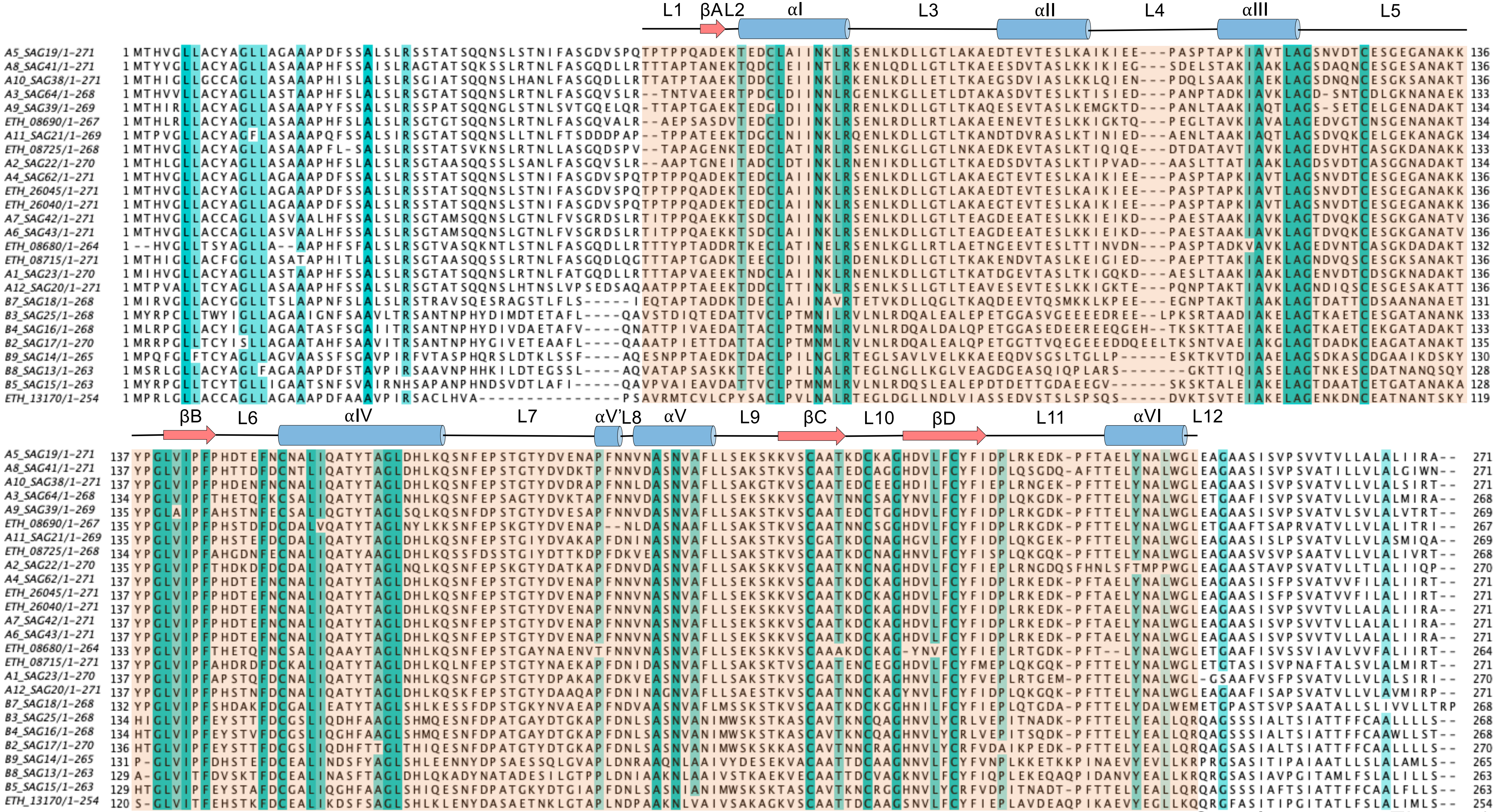

Supplementary Figure 1

Supplementary Figure 1:

Multiple sequence alignment of the 26 *E. tenella* SAG^B members. Residues that are represented in the EtSAG19 structure are shaded in orange to reflect the colour scheme of figure 2, while the 37 residues identified as being conserved in > 95 % of the $EtSAG^B$ sequences are highlighted in blue. The positions of the elements of secondary structure and inter-connecting loops of EtSAG19 are shown above the sequences. Residue numbers are presented at the beginning and end of each line. The ID for each EtSAG member is that used in Reid *et al.*, 2014[12]. Figure generated using Jalview[52].

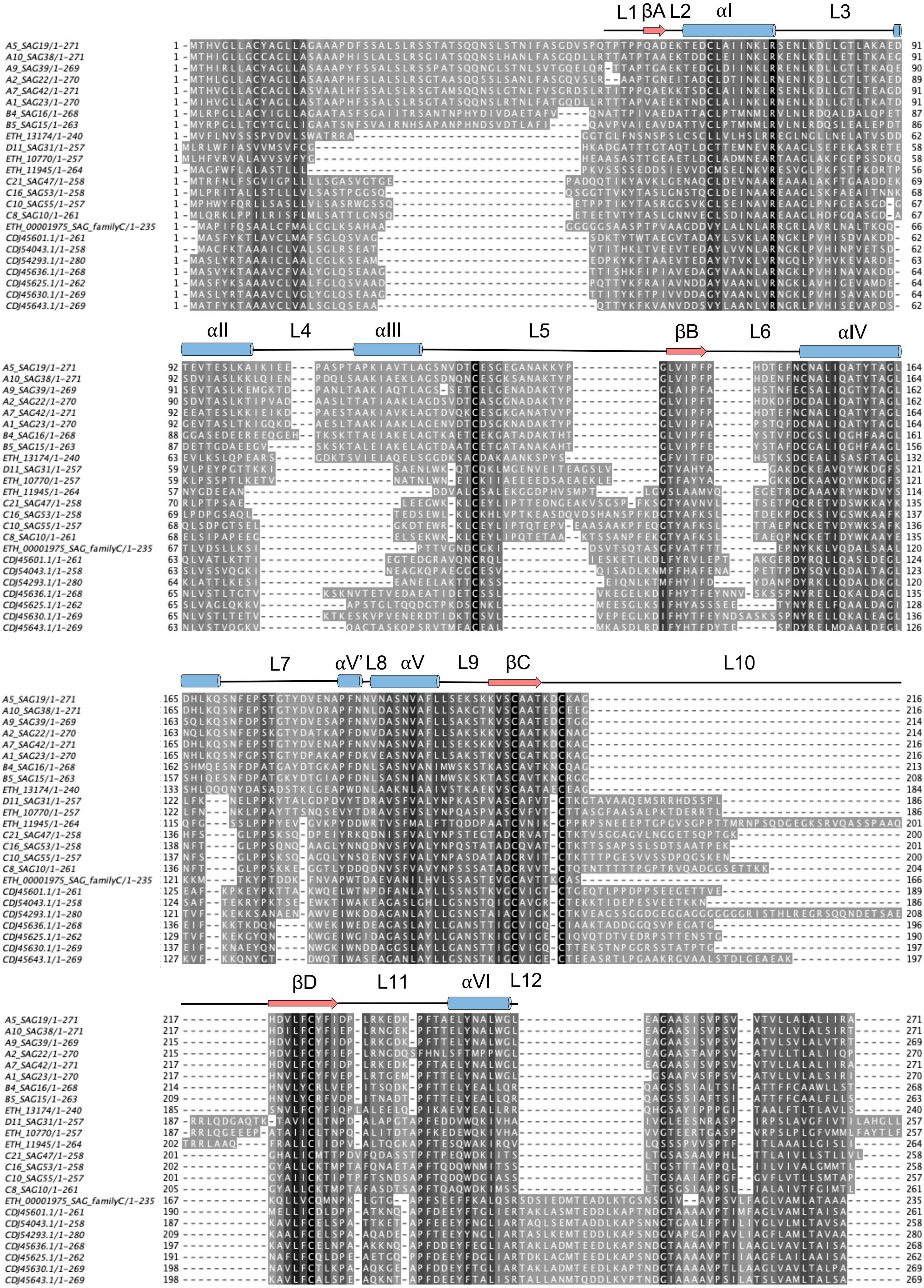

Supplementary Figure 2

Supplementary Figure 2:

Multiple sequence alignment of eight representative members from each EtSAG family (1-8 SAG^A, 9-16 SAG^B, 17-24 SAG^C). The positions of the elements of secondary structure and inter-connecting loops of EtSAG19 are shown above the sequences. Residue numbers are presented at the beginning and end of each line. The ID for each EtSAG member is that used in Reid *et al.*, 2014[12]. Figure generated using Jalview[52].

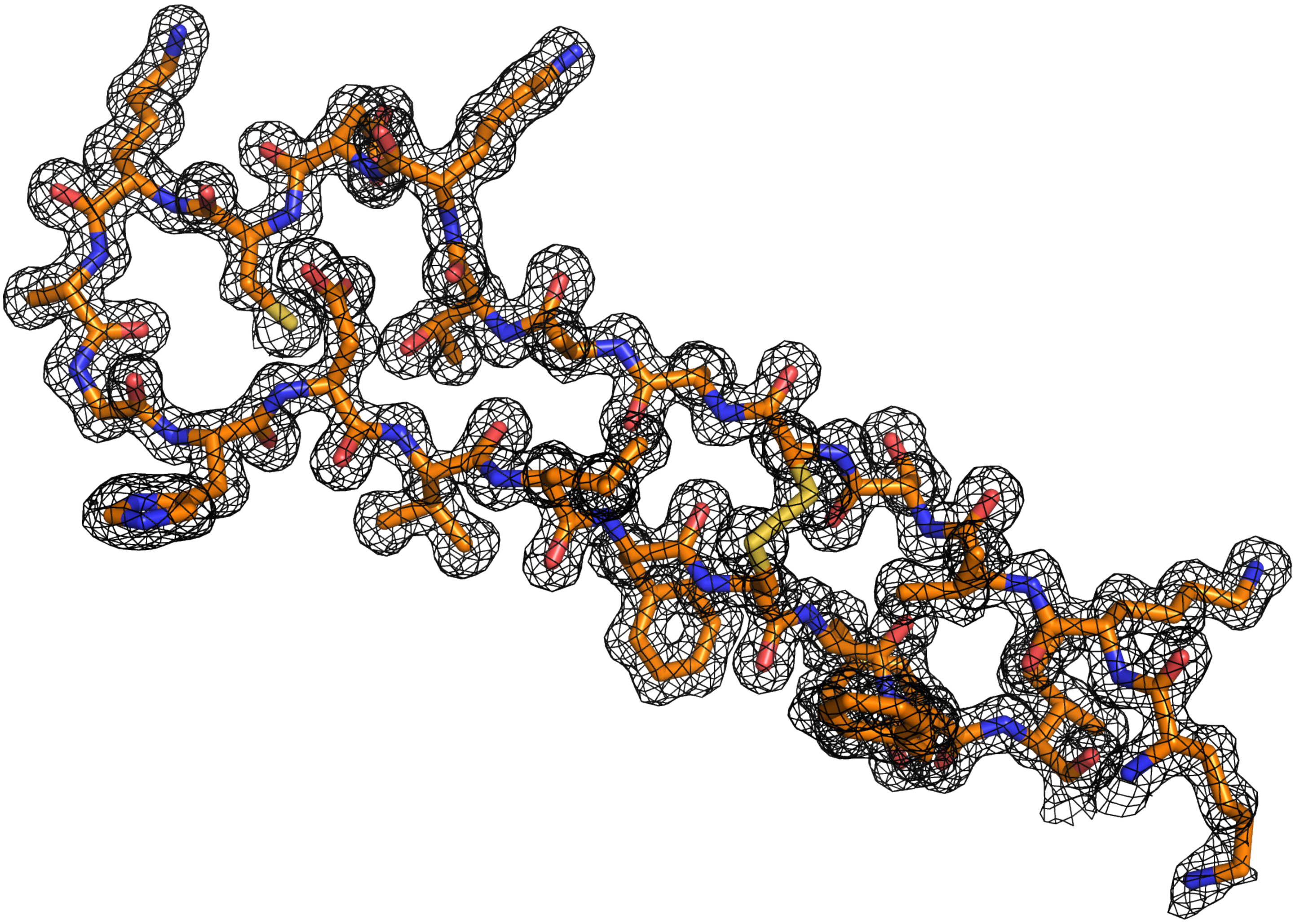

Supplementary Figure 3

Supplementary Figure 3:

Representative electron density of the final 2Fo-Fc map around β-strands C and D from the EtSAG19 structure contoured at 1.0 sigma.

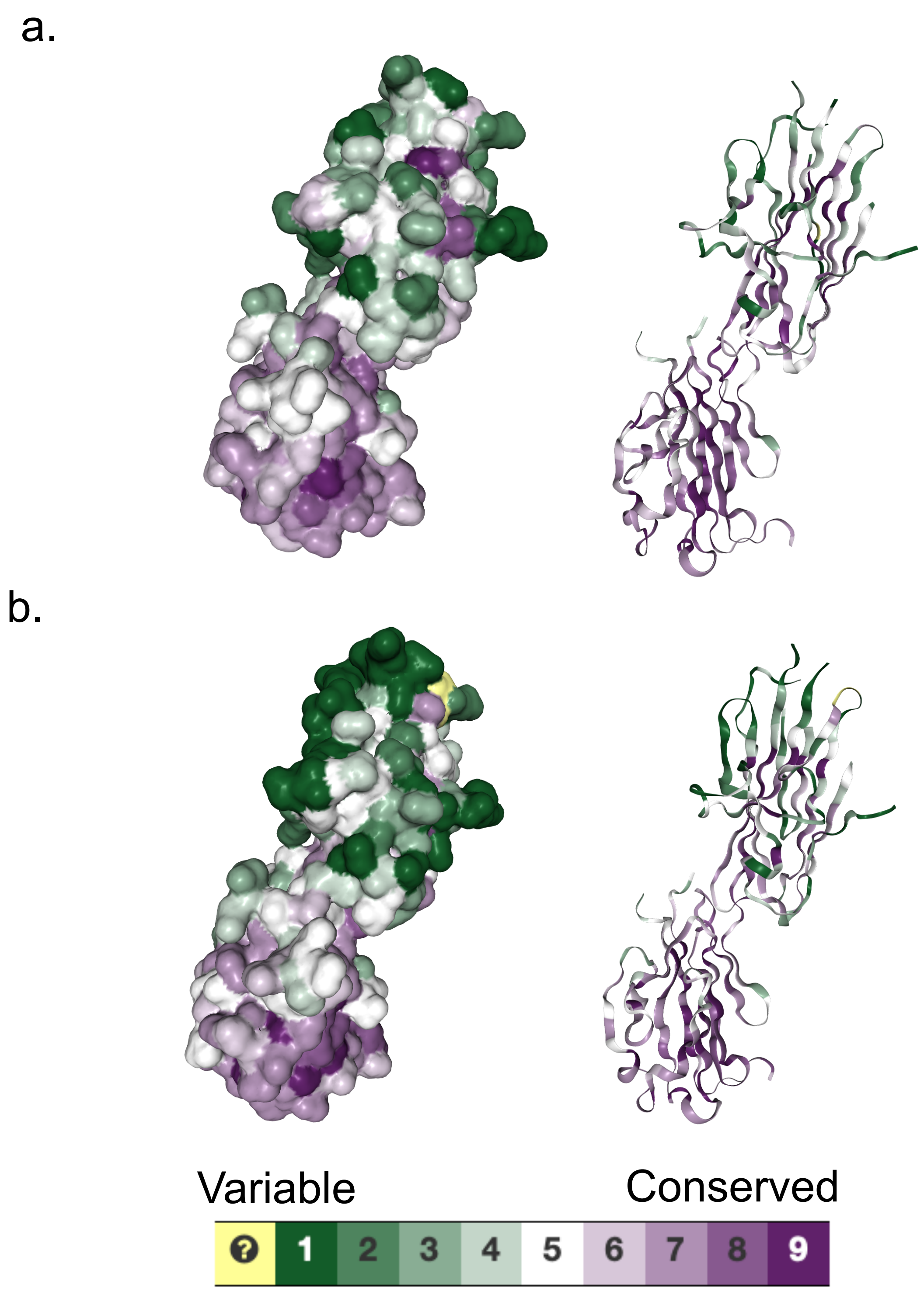

Supplementary Figure 4

Supplementary Figure 4:

Sequence conservation at the surface of members of the *B. besnoiti* and *N. caninum* SAG superfamily. Surface (left) and cartoon (right) representations to illustrate the pattern of sequence conservation and diversity in members of the SAG superfamily for **a**) *B. besnoiti* and **b**) *N. caninum* based on the Consurf analysis using the structure of the homologous *T. gondii* BSR4 protein as a guide. The figures are coloured using the colour blind Consurf colour representation.

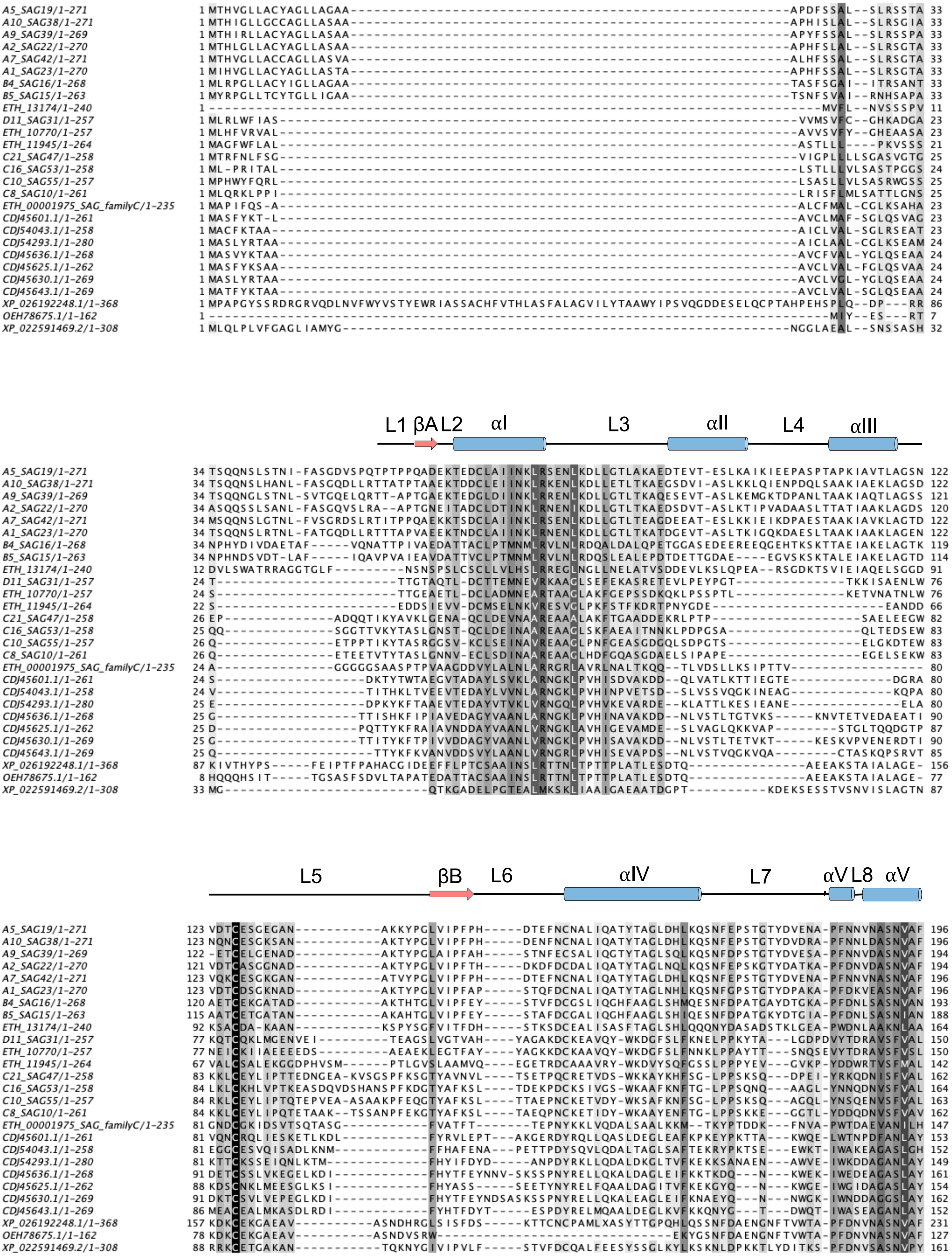

Supplementary Figure 5

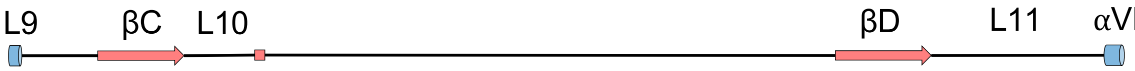


```
A5_SAG19/1-271                197 LLSEKSKKVSCAATKDCKAGH----------------------------------------------DVLFCYFIDPLRKE-D-KPFTAEL 239
A10_SAG38/1-271               197 LLSAKGTKVSCAATEDCEEGH----------------------------------------------DILFCYFIEPLRNG-E-KPFTTEL 239
A9_SAG39/1-269                195 LLSAKSTKVSCAATEDCTGGH----------------------------------------------DVLFCYFIDPLRKG-D-KPFTTEL 237
A2_SAG22/1-270                195 LLSAKSKKVSCAATKNCNAGH----------------------------------------------DVLFCYFIEPLRNG-D-QSFHNLS 237
A7_SAG42/1-271                197 LLSEKSKKVSCAATKDCKAGH----------------------------------------------DVLFCYFIDPLRKE-D-KPFTAEL 239
A1_SAG23/1-270                197 LLSAKSTKVSCGATKDCKAGH----------------------------------------------NVLFCYFVEPLRTG-E-MPFTTEL 239
B4_SAG16/1-268                194 IMWSKSTKASCAVTKNCQAGH----------------------------------------------NVLYCRLVEPITSQ-D-KPFTTEL 236
B5_SAG15/1-263                189 IMWSKSKTASCAVTKNCRGGH----------------------------------------------NVLYCRFVDPITNA-D-TPFTTEL 231
ETH_13174/1-240               165 IVSTKAEKVSCAATAECEAGS----------------------------------------------NVLFCYFIQPLALE-ELQPIKAEV 208
D11_SAG31/1-257               151 YNPKASPVASCAFVTCTKGTAVAAQE---------------------MSRRHDSSPLRRLQDGAQTKTAVICLTNPDALAP-GTAPFEDDV 219
ETH_10770/1-257               151 YNPQASPVASCVFVTCTTASGFAASA---------------------LPKTDERRTLRRLQGEEEPATAIICLTNPQALIT-DTAPFKEDE 219
ETH_11945/1-264               143 FTTQDDPAATCVNIKCPPRPSNEEEPTGPGVSGPPTTMRNPSQDGEGKSRVQASSPAAQTRRLAAQFRALLCIIDPVALTQ-GKAPFTESQ 232
C21_SAG47/1-258               163 YNPSTEGTADCRVATCTKTVSGGAG----------------------------VLNGGETSQPTGKGHALICMTTPDVFQDASSTPFTPEQ 225
C16_SAG53/1-258               165 YNPQSTATADCQVATCTKTTSSAPS-----------------------------SLSDTSAATPEKGYALLCKTMPTAFQNAETAPFTQDQ 226
C10_SAG55/1-257               164 YNPASDATADCRVITCTKTTTPGESVVSS-----------------------------DPQGSKENGYAIICKTIPTPFTSNDSAPFTQDQ 225
C8_SAG10/1-261                163 YNPSSSATADCRVVTCTQTNTTTTTPGPTRVQAD------------------------GGSETTKKGYALLCKTMPTAFASDTSAPFTQAQ 229
ETH_00001975_SAG_familyC/1-235 148 VLSSASTEVGCAVTTK-----------------------------------------------CASKQLLVCQMNPKLG---TGAPFSEEF 188
CDJ45601.1/1-261              154 LLSSNSTKVGCVIGTCTGEQTLPPDPP------------------------------SEEGETTVEMELLICDLDPPATK--NQAPFDEEY 212
CDJ54043.1/1-258              153 LLGSNSTQIGCAVGRCTEKKTIDEP--------------------------------ESVEETKKNKAVLFCELSPATTK--ETAPFEEEY 209
CDJ54293.1/1-280              150 LLGSNSTAIACVIGKCTKVEAGSSGGDGEGGAGG-------GGGGGRISTHLREGRSQQNDETSAEKAALFCELSPAAQA--DEAPFTEEY 231
CDJ45636.1/1-268              162 LLGANSTTIGCVIGQCIAAKTADDG-G------------------------------QSVPEGATGKAVLFCELNPAAKK--NQAPFDDEY 219
CDJ45625.1/1-262              155 LLGANSTKIGCVIGECIQVQTDTVEDR------------------------------PSTTENSTGNAFLFCQLDPEAET--GQPPFDEEY 213
CDJ45630.1/1-269              163 LLGANSTTIGCVIGQCTTEKSTNPG-G------------------------------RSSTATPTGNAVLFCTLKPAAQK--GEAPFDDEY 220
CDJ45643.1/1-269              153 LLGSNSTKIGCVIGECTEEASRTLPGAAKRGV---------------------AALSTDLGEAEAKKAVLFCALSPEAQK--NTAPFDEEY 220
XP_026192248.1/1-368          232 VMWNGNAKVSCVWSTDCSEAH----------------------------------------------NVLXC-AFEAEIQD-GAQPFLSEL 274
OEH78675.1/1-162              128 VMWNGVNSTTLFCSAKLARRL------------------------------------------QWPLASLLCFSFSY-------------- 162
XP_022591469.2/1-308          162 MISTQDNQVSCAVTNNCDAGR----------------------------------------------NVLYYAFKTNLKSK-E--PFRWGF 203
```

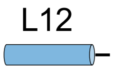


```
A5_SAG19/1-271                240 Y-NALWGLEAGAA----------------SISVPSVVTVLLALALIIRA------------------------------------------ 271
A10_SAG38/1-271               240 Y-NALWGLEAGAA----------------SISVPSVATVLLVLALSIRT------------------------------------------ 271
A9_SAG39/1-269                238 Y-NALWGLEAGAA----------------SISVPSVATVLSVLALVTRT------------------------------------------ 269
A2_SAG22/1-270                238 FTMPPWGLEAGAA----------------STAVPSVATVLLTLALIIQP------------------------------------------ 270
A7_SAG42/1-271                240 Y-NALWGLEAGAA----------------SISVPSVVTVLLALALIIRA------------------------------------------ 271
A1_SAG23/1-270                240 Y-NALWGLGS-AA----------------FVSFPSVATVLLVLALSIRI------------------------------------------ 270
B4_SAG16/1-268                237 Y-EALLQRQAGSS----------------SIALTSIATTFFCAAWLLST------------------------------------------ 268
B5_SAG15/1-263                232 Y-EALLQRQAGSS----------------SIAFTSIATTFFCAALFLLS------------------------------------------ 263
ETH_13174/1-240               209 Y-EALLRRQHGSA----------------YIPPPGITAALFTLTLAVLS------------------------------------------ 240
D11_SAG31/1-257               220 W-QKIVHAIVGLEESNRA----------SPIRPS--LAVGFIVTILAHGLL---------------------------------------- 257
ETH_10770/1-257               220 W-QKIVHAVVGTEERTGA----------SPVRPS--LPLGFVMMLFAYTLF---------------------------------------- 257
ETH_11945/1-264               233 W-AKIRQVLQSSS----------------PVVSP--TFITLAAALLGISLI---------------------------------------- 264
C21_SAG47/1-258               226 W-DKIITSLTGSA----------------SAAVP--HLTTLAIVLLSTLLVL--------------------------------------- 258
C16_SAG53/1-258               227 W-NMITSSLTGST----------------TTAVP--QLIILVIVALGMMTL---------------------------------------- 258
C10_SAG55/1-257               226 W-DGIASSLTGSA----------------AIAFP--GFGVFLVTLLSMTAP---------------------------------------- 257
C8_SAG10/1-261                230 W-DKIMSSLTGSG----------------SIAAP--SLIALAIVTFGIMTL---------------------------------------- 261
ETH_00001975_SAG_familyC/1-235 189 F-KALQSRSDSIEDMTEADLKTGSNSGIV--AVPSVLFAGLVAMLATAAA----------------------------------------- 235
CDJ45601.1/1-261              213 F-TGLIERTAKLASMTEDDLKAPTNDGTAAAAVPTIMFAGLVAMLTAISA----------------------------------------- 261
CDJ54043.1/1-258              210 F-NGLIARTAQLSEMTADDLKAPSNNGTGAALFPTILIYGLVLMLTAVSA----------------------------------------- 258
CDJ54293.1/1-280              232 Y-NGLIARTASLKEMTEDDLKAPSNDGVAPGAIPAVLIAGFLAMLTAVAA----------------------------------------- 280
CDJ45636.1/1-268              220 F-EGLIARTAKLADMTEEDLKAPSNDGTAAAAVPTILAAGLVAVLTAISA----------------------------------------- 268
CDJ45625.1/1-262              214 F-DGLIARTDKLAGMTEEDLKAPSNDGTAAAAVPTIIAAGFLAILAAISA----------------------------------------- 262
CDJ45630.1/1-269              221 F-TGLIARTAKLADMTEEDLKAPTNDGTAAAAVPTLLAAGLVAVLTALPA----------------------------------------- 269
CDJ45643.1/1-269              221 F-TALISRTALLANMTQDDLKVPTNDGIAAAAVPSILATSLVAMLTALSA----------------------------------------- 269
XP_026192248.1/1-368          275 Y-NALLQRQAGSA----------------SAVAPGITAHLPER-LRPSALYYPFVLGIESNSDSNNSYTLPCSLFILGVLMAGRIPAGRFI 347
OEH78675.1/1-162                  -------------------------------------------------------------------------------------------
XP_022591469.2/1-308          204 ----LLDRKASAQ----------------KQQVKQVISYLFNHRYSPPILQKVSFRCFAVDRVSQFCPLSSALSPSAKSGRELGDQPVHCH 274
```

```
A5_SAG19/1-271                    ----------------------------------
A10_SAG38/1-271                   ----------------------------------
A9_SAG39/1-269                    ----------------------------------
A2_SAG22/1-270                    ----------------------------------
A7_SAG42/1-271                    ----------------------------------
A1_SAG23/1-270                    ----------------------------------
B4_SAG16/1-268                    ----------------------------------
B5_SAG15/1-263                    ----------------------------------
ETH_13174/1-240                   ----------------------------------
D11_SAG31/1-257                   ----------------------------------
ETH_10770/1-257                   ----------------------------------
ETH_11945/1-264                   ----------------------------------
C21_SAG47/1-258                   ----------------------------------
C16_SAG53/1-258                   ----------------------------------
C10_SAG55/1-257                   ----------------------------------
C8_SAG10/1-261                    ----------------------------------
ETH_00001975_SAG_familyC/1-235    ----------------------------------
CDJ45601.1/1-261                  ----------------------------------
CDJ54043.1/1-258                  ----------------------------------
CDJ54293.1/1-280                  ----------------------------------
CDJ45636.1/1-268                  ----------------------------------
CDJ45625.1/1-262                  ----------------------------------
CDJ45630.1/1-269                  ----------------------------------
CDJ45643.1/1-269                  ----------------------------------
XP_026192248.1/1-368          348 VCPQRALFTDIEGQYPAIQDF------------- 368
OEH78675.1/1-162                  ----------------------------------
XP_022591469.2/1-308          275 CPGILRRSVHLLLSPWHAPGVGKALQRPFRLFLA 308
```

Supplementary Figure 5

Supplementary Figure 5:

Multiple sequence alignment of the three putative SAG representatives from *C. cayetanensis* with the 24 representative members from *E. tenella* and *E. brunetti*. Indicating a similar pattern of insertion and deletions, located distal from the GPI anchor. The positions of the elements of secondary structure and inter-connecting loops of EtSAG19 are shown above the sequences. Figure generated using Jalview[52].

Supplementary Table 1 - Hydrogen bonding distance found in the N72xxR75 motif.

Hydrogen bond	Distance (Å)
(N72) OD---N (L86)	2.77
(N72) ND---O (L86)	3.03
(R75) NE---O (G84)	2.77
(R75) NH1---O (S199)	2.87
(R75) NH1---O (L197)	2.81
(R75) NH2---O (S199)	3.12
(R75) NH2---O (G84)	3.04

Supplementary Table 2 – Core residues from the CAP superfamily representatives superimposed with the equivalent residues in EtSAG19**.**

	α-helices				β-strand			Rmsd (Å)
	I	II	III	IV	B	C	D	
EtSAG19	67-76	110-116	152-161	191-199	139-145	202-211	218-228	
GAPR-1	13-22	38-44	76-85	101-199	65-71	112-121	128-137	1.07
Ves-v-5	44-53	86-92	126-135	154-162	111-117	165-174	184-193	1.09
Na-ASP2	15-24	57-63	96-105	127-135	80-86	138-147	153-162	1.17
