# Supplementary material for: The structure of a major surface antigen SAG19 from Eimeria tenella unifies the Eimeria SAG family
Source: Commun Biol. 2021 Mar 19;4:376. doi: 10.1038/s42003-021-01904-w (PMC7979774; doi:10.1038/s42003-021-01904-w)
Supplement: Supplementary file 2 — Description of Additional Supplementary Files [file 42003_2021_1904_MOESM2_ESM.pdf]

Description of Additional Supplementary Files

File Name: Supplementary Data 1

Description: FASTA sequences of the 89 putative *E. tenella* SAG proteins identified from genome sequencing studies[12].

File Name: Supplementary Data 2

Description Multiple sequence alignment of the 79 *E. tenella* SAG^A and SAG^B protein sequences aligned as part of this study.

File Name: Supplementary Data 3

Description: Multiple sequence alignment of the 118 E. tenella and E. brunetti SAG protein sequences aligned as part of this study.

File Name: Supplementary Data 4

Description: Multiple sequence alignment of the 66 *E. tenella*, *E. brunetti and E. mitis* SAG protein sequences aligned as part of this study.
